# Examining the Effects of Ambient Temperature on Pre-Term Birth in Central Australia

**DOI:** 10.3390/ijerph14020147

**Published:** 2017-02-04

**Authors:** Supriya Mathew, Deepika Mathur, Anne B. Chang, Elizabeth McDonald, Gurmeet R. Singh, Darfiana Nur, Rolf Gerritsen

**Affiliations:** 1 Northern Institute, Charles Darwin University, Ellengowan Dr., Casuarina, NT 0810, Australia; deepika.mathur@cdu.edu.au (D.M.); rolf.gerritsen@cdu.edu.au (R.G.); 2 Menzies School of Health Research, Rocklands Drive, Casuarina, NT 0810, Australia; Anne.Chang@menzies.edu.au (A.B.C.); Elizabeth.McDonald@menzies.edu.au (E.M.); Gurmeet.Singh@menzies.edu.au (G.R.S.); 3 School of Computer Science, Engineering and Mathematics, Flinders University, Adelaide, SA 5001, Australia; darfiana.nur@flinders.edu.au

**Keywords:** indigenous, climate change, preterm birth, arid, desert, remote

## Abstract

Preterm birth (born before 37 completed weeks of gestation) is one of the leading causes of death among children under 5 years of age. Several recent studies have examined the association between extreme temperature and preterm births, but there have been almost no such studies in arid Australia. In this paper, we explore the potential association between exposures to extreme temperatures during the last 3 weeks of pregnancy in a Central Australian town. An immediate effect of temperature exposure is observed with an increased relative risk of 1%–2% when the maximum temperature exceeded the 90th percentile of the summer season maximum temperature data. Delayed effects are also observed closer to 3 weeks before delivery when the relative risks tend to increase exponentially. Immediate risks to preterm birth are also observed for cold temperature exposures (0 to –6 °C), with an increased relative risk of up to 10%. In the future, Central Australia will face more hot days and less cold days due to climate change and hence the risks posed by extreme heat is of particular relevance to the community and health practitioners.

## 1. Introduction

Preterm birth (born before 37 completed weeks of gestation) is one of the leading causes of death among children under 5 years of age [1]. Preterm births can result in ongoing health challenges during childhood and over the course of life [2]. Extreme prematurity frequently requires intensive medical management over many weeks to months. This causes families stress concerning the health and welfare of their infant [3]. It leads to disruptions in family life and unplanned expenses for families. Also, the cost to the health care system to provide medical management for premature infants is high [4,5]. 

In 2013, around 9% of Australian babies were born preterm [6]. Risks for spontaneous preterm delivery include having more than one foetus, inherited traits, low socio-economic status, lifestyle (use of alcohol, tobacco and other drugs), and general health conditions (nutritional status, infection and chronic disease, e.g., diabetes and hypertension) [7]. Risks also are increased for women who are: Indigenous (14% compared to 8% for non-Indigenous mothers), as well as young (<20 years) and old mothers (>40 years) (10% to 12% compared with 8% of mothers aged 20–39) [8]. Mothers who reside in very remote locations (13% compared to 8% for those who live in urban centres) are also at risk of having premature births. In Australia, the Northern Territory (NT) (9.1%) and Tasmania (9.6%) have the highest preterm birth rates [8]. 

In addition to the factors above, environmental parameters such as temperature, humidity, ozone, carbon monoxide, nitrous oxide and particulate matter may also be important factors affecting preterm birth and have been the focus of recent studies [9,10,11]. However, findings from international studies on the effects of maternal exposure to heat stress on preterm delivery are variable. While some studies report a significantly higher risk of preterm birth with an increase in ambient temperature [9,12,13,14,15,16,17], other studies report no significant associations [18,19,20,21]. It is biologically plausible that increased ambient temperature leads to an increased risk of preterm delivery due to changes in the thermoregulatory capacities of pregnant women caused by their increased body mass beyond 20 weeks of gestation and increased metabolic rates [9,22]. A further plausible reason suggested points to changes in haemodynamics (dehydration, hyperthermia) and/or uterine activity in pregnant women when they are exposed to short term high temperature [23]. 

Global model simulations indicate that the number of comfortable thermal days is likely to decline due to atmospheric warming in many parts of Australia [24]. The heat effects are likely to be exacerbated in regions with extreme heat for prolonged periods of time such as in Central Australia. Thus, it is important to identify if there is an association between heat and preterm birth in Central Australia. 

In this paper, we evaluated whether high ambient temperature increases the risk of preterm birth in a Central Australian town of the Northern Territory. We hypothesised that extreme heat conditions in Central Australia could exert maternal stress and thus there is likely to be an association between maximum temperatures and preterm births for this region. 

## 2. Materials and Methods 

In the Central Australian region, the majority of communities are classified as “remote” or “very remote” locations mainly due to their distances from essential services. In this paper we focus on Alice Springs, Central Australia’s major town that serves a population of 25,186 people, of which 19% are Indigenous [25]. The male to female ratio is almost equal among the non-Indigenous population, while the percentage of females (53%) is slightly higher than the percentage of males among the Indigenous population [25]. 

Alice Springs has a semi-arid climate, with a low and variable rainfall. The annual rainfall and average temperature for the region is around 260 mm and 29.5 °C (average of the past 10 years according to the Bureau of Meteorology Alice Springs airport station data) respectively. Climate projections indicate a rise in temperature for this region in the range of 1.6–7 °C by 2100, as well as an increase in the number of hot days and a decrease in the number of cold nights [26]. The annual average number of days over 35 °C in Alice Springs is likely to increase from a current value of 89 to somewhere between 96 and 125 days by 2030 [27]. The projection of higher average maximum temperatures is of immense concern as this location already has more than three months of the year with daytime temperatures regularly above 35 °C. 

All pregnant women living in remote communities (mostly Aboriginal) in the Central Australia region are advised to move to Alice Springs from around 34 weeks to ensure safe delivery. The Alice Springs Hospital (ASH) is the only obstetric services provider in Central Australia. Our analysis is confined to Alice Springs (Alice Springs Hospital) to avoid the effects of spatial variation in temperature within Central Australia. 

In addition to temperature, there are other environmental variables such as air pollutants and humidity that have an effect on preterm birth [9]. In this study, analysis was restricted to minimum and maximum daily temperature as humidity is considered to be of lower importance to desert climates and air pollution data was not available for the region. Temperature records from the Alice Springs airport station (Bureau of Meteorology station 15590, downloaded via http://www.bom.gov.au/climate/data/) were used for the analysis (see Table S1 for a summary of temperature data for Alice Springs). 

The NT Department of Health, Health Gains Planning branch holds information on all births within the NT (NT Midwives Data Collection). Data for the Central Australian region was obtained from the NT Department of Health for the purpose of this study. Ethics approval to access the data and conduct this research was obtained from the Central Australian Human Research Ethics Committee (CAHREC) and the reference number is HREC-15-326. This data collection consisted of 26460 patient records spanning the period from 1 January 1986 to 31 December 2013. Preterm birth was defined as birth before 37 weeks of gestation. Total births considered for the temperature effects analysis were restricted to live singleton births at the Alice Springs Hospital during the period of 1986–2013. Elective caesareans and data records with missing date of births and gestational age were excluded. Emergency caesareans and induced labour were included as the dataset did not contain details on the reasons for these. Data items obtained included information on maternal age, alcohol and smoking status, history of certain diseases such as STI (sexually transmitted diseases), UTI (urinary tract infection), cardiac disease, anaemia, epilepsy, gestational diabetes, renal disease and syphilis, pre-existing maternal conditions such as diabetes and hypertension, plurality, gestation age, Indigenous status, place of residence and birth, gender of baby and birth weight. 

A Poisson Generalised Additive Model was used to account for over-dispersion to examine the short-term effects of temperature on the risk of preterm birth [28]. Distributed lag non-linear models were applied to permit non-linearity and simultaneously take into account the potential delayed effect of temperature exposure [29]. Lag-specific relative risks (RR) of preterm birth are discussed for different temperature values (50th, 90th and 99th percentiles) for the whole data period using median temperature of the temperature records as a reference. A time window of up to 21 days before delivery was considered. The association between temperature (maximum and minimum) and the relative risk of preterm birth is examined for the whole data period (1986–2013). 

Following [28], a flexible approach consisting of the introduction of a natural cubic spline function in the variable dimension of the cross-basis was selected. Similar functions were introduced to flexibly model the lag structure up to 21 days. Based on the residual deviance, 5 degrees of freedom (df) with degree 3 were introduced into the quadratic B-spline function of the temperature relationships. The knots were placed at equally spaced values in the log-scale of the lags [29]. Long-term trends and seasonality (see Figure S2) were controlled by including a penalized cubic spline function with degrees of freedom 0.8 and 1 per month and/or year, respectively in each term. In addition, factor variables such as the day of the week and holidays were introduced into the model. Residual deviances determined which terms to include in the final model.

The pregnancies at risk model [28], originated from the distributed nonlinear lag models (DNLM) [29] is given by:

$$\log\left(\frac{N_{i}}{Z_{i}}\right)=\beta_{0}+\beta_{1}X_{1}+\cdots +\beta_{p}X_{p}+\overset{q}{\underset{j=p+1}{\sum }}s_{j}\left(X_{j},\gamma_{j}\right)+\beta_{w}\log\left(W_{i}\right)$$

where $N_{i}$ is the daily preterm counts, $Z_{i}$ is the pregnancies at risk of preterm birth on a given day i, $W_{i}$ (gestational weight) is the sum of the probabilities of giving birth conditional on the gestational age of pregnancies at risk on a given day i. Increasing gestational age increases the chances of delivery and hence $\frac{N_{i}}{Z_{i}}$ is modelled to address the issue [16,30]. The functions $s_{j}$ denote smoothed relationships between the explanatory variables $X_{j}$ and the linear predictor defined by the parameters $\gamma_{j}$.

In this study, a 22-week window (the difference between the maximum gestational age in the data and the minimum gestational age for preterm birth) is used to calculate the pregnancies at risk counts. 

## 3. Results

After applying the exclusion criteria to the dataset obtained from the Department of Health, the dataset included 16,870 single natural births that took place during the period of 1986–2013, with a mean daily count of 1.65 births per day (see Figure S2). The data included 1401 preterm births, which comprised 8.3% of the data used (see Table S2).

In the Poisson Generalised Additive Model run with data of both Indigenous and non-Indigenous pregnant mothers, maximum temperature and minimum temperature were statistically significant parameters (significance level of 5%). The same model was run after separating the data for Indigenous and non-Indigenous mothers. Daily maximum temperature and age of mother were significant (significance level of 5%) when the model was run on Indigenous data. Maximum temperature, anaemia, gestational diabetes and maternal age were significant (significance level of 5%) among the data for non-Indigenous mothers. Smoking status and alcohol status were also significant, but these variables were dominated by the option ‘not stated’. The results have been mainly focused on the whole data set, as the sample size is considerably reduced when the data sets are separated, especially for the Indigenous population. 

### Effects of Maximum Temperature and Minimum Temperature on Preterm Deliveries in Central Australia 

**Figure 1:**
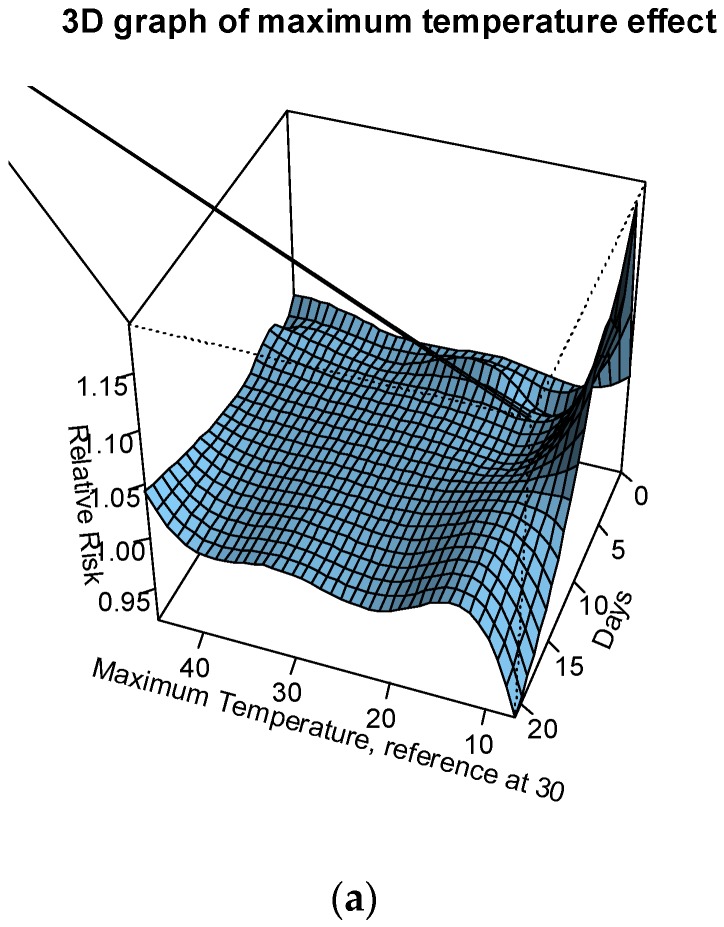

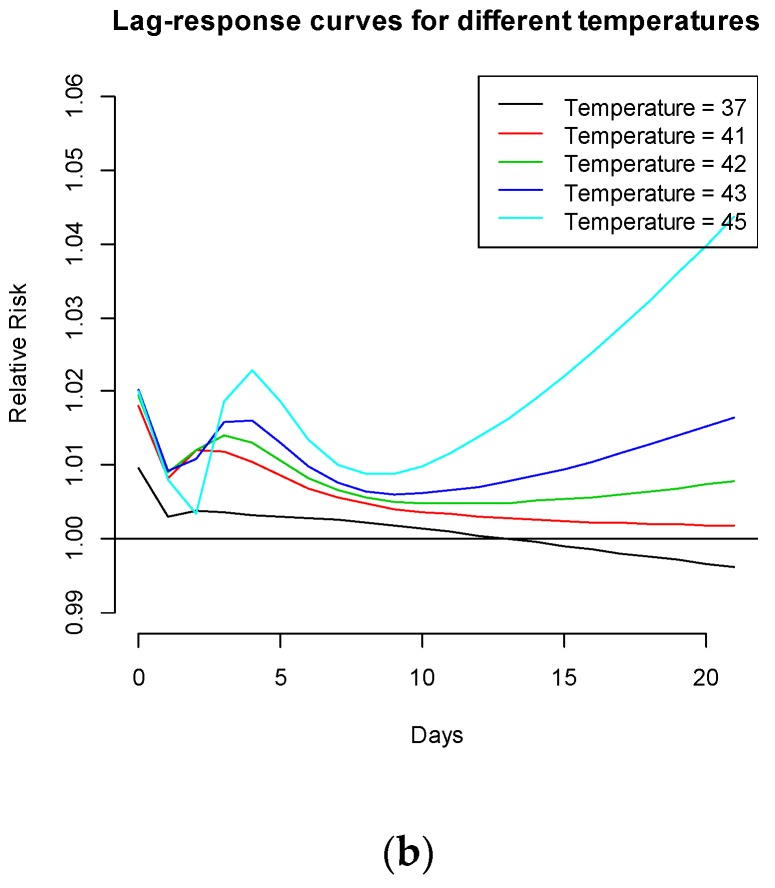
(**a**) Three-dimensional graphs of the relative risk (RR) of preterm birth to the exposure of maximum temperature and (**b**) RR of preterm birth to the exposure of maximum temperatures (median, P90, P95, P99 and maximum temperature recorded during the summer periods in 1986–2013). The median maximum temperature value of 30 °C is used as a reference and lags up to 21 days are plotted.

**Figure 2:**
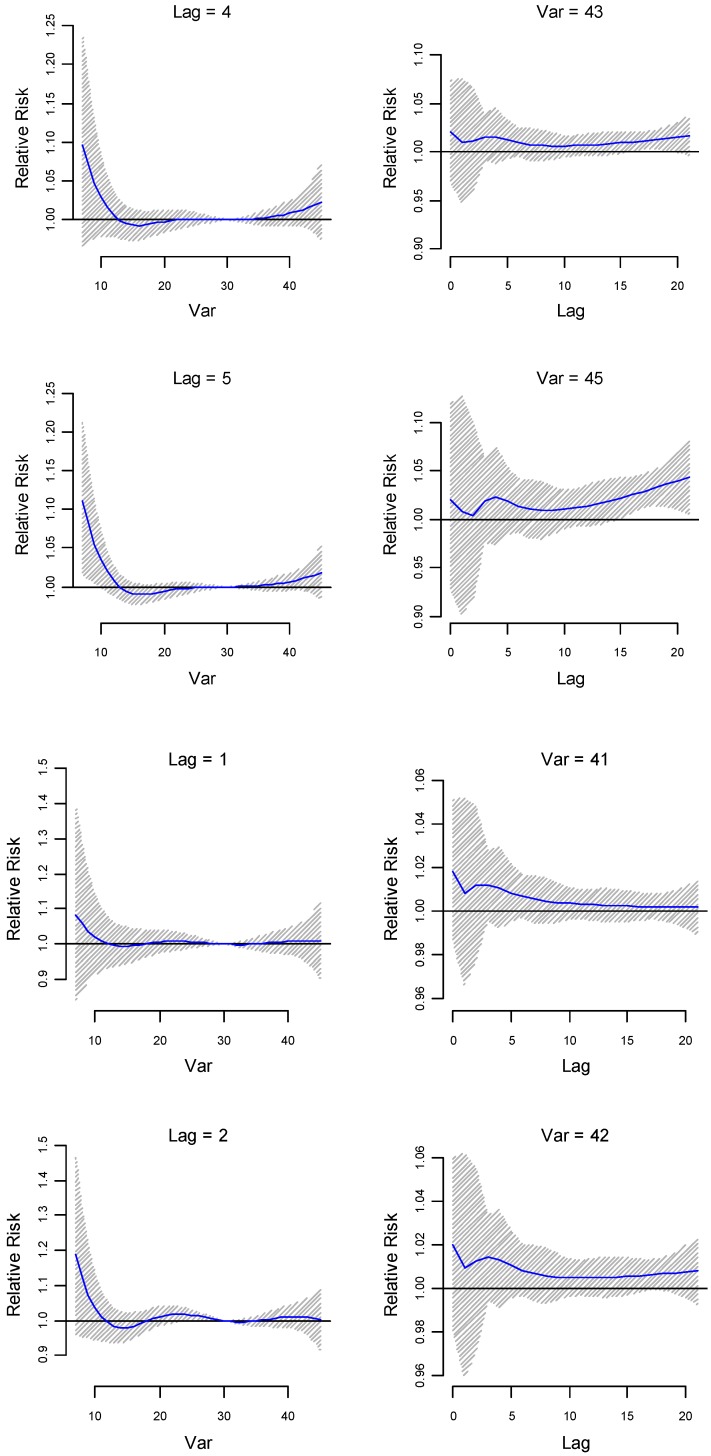
Lag-specific (at lags 1, 2 and 4, 5) relative risk (outcome) estimates with their 95% confidence interval plotted for different maximum temperatures. The median value of the whole temperature annual series (30 °C) is used as a reference.

The association between both maximum and minimum temperature with preterm birth was modelled for up to 3 weeks before delivery. The relative risks (RR) of preterm birth were assessed against the following temperatures: the median of summer season at 37 °C; the 90th percentile of the summer season (41 °C); the 95th percentile of the summer season (42 °C); the 99th percentile of the summer season (43 °C); and the maximum temperature recorded (45 °C). The median temperature of the whole temperature record (30 °C) is used as the reference temperature. The relative risk of preterm birth was greater than 1 from temperatures 41 °C to 45 °C (see Figure 1a,b and Figure 2). An immediate increase in RRs (day of delivery) of up to 2%, a slightly delayed increase in RRs (within 1 week before delivery) of up to 2.5%, and a delayed exponential increase in RRs (2–3 weeks before delivery) of up to 4.5% were observed for temperatures >41 °C.

The lag-specific relative risk estimates (at lower lags 1, 2, 4 and 5) for different temperatures (41 °C, 42 °C, 43 °C and 45 °C) are plotted in Figure 2. The 95% confidence interval (CI) of the RR to preterm birth at the maximum temperature of 45 °C is above 1 at lags 15 to 20, that is around 2 weeks before delivery, which indicates delayed effects of maximum temperature. The same model run for the Indigenous population indicates not immediate, but slightly delayed (around 2 days before delivery) and higher relative risks (1%–3.5%) (see Figure S3a). Increased delayed relative risks were observed around 2 weeks before delivery for temperatures ≥41 °C. Beyond 2 weeks before delivery, there was an exponential decrease in the relative risks. For non-Indigenous pregnant mothers, immediate increased RRs (>6%) and slightly delayed increased RRs (~2.5%) are observed for temperatures between 41 °C and 43 °C (see Figure S3b). Around 2 weeks before delivery, relative risks seem to increase by up to 4%, but thereafter risks seem to remain constant or slowly decrease, unlike the exponential increase observed in Figure 1b. 

The cumulative effects of exposure to maximum temperatures greater than 40 °C were statistically significant (Figure S4a). An effect of the exposure to the temperature at 40 °C cumulatively up to 21 days before delivery was observed with an increased risk of preterm birth up to 8.3% with the 95% CI of 1.03 to 1.15. Cumulative effects for the non-Indigenous population followed a similar pattern to that of the combined population (Figure S5b). The cumulative effects of temperature exposure among Indigenous pregnant mothers were quite different. The relative risk can increase up to 12% for temperatures between 30 and 38 °C, but seem to decline for higher temperatures (see Figure S5a).

**Figure 3:**
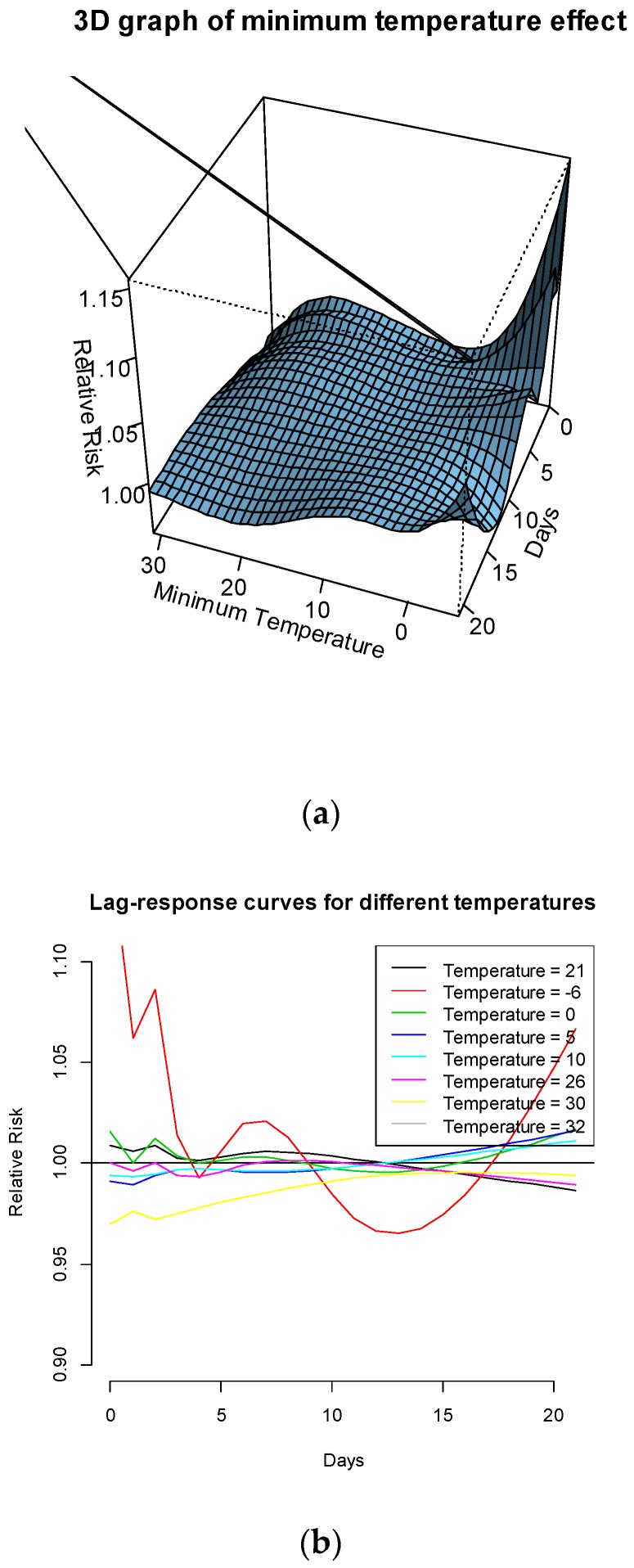
(**a**) Three-dimensional graph of the RR of preterm birth to the exposure of minimum temperature and (**b**) RR of preterm birth for the exposure to different minimum temperatures (Minimum = –6 °C, Median = 21 °C, P90 = 26 °C, P99 = 30 °C and the maximum = 32 °C of minimum temperatures), using the median minimum temperature value at 14 °C of the whole annual series as a reference.

The association of minimum temperature with preterm birth is also modelled. The RR of preterm birth is peaked at minimum temperatures at around 0 °C at some lower and higher lags. There seem to be lower RRs at other minimum temperatures (see Figure 3a). Figure 3b plots the risks of preterm birth at the following temperatures: the median of minimum temperatures in summer season (21 °C); the 90th percentile (26 °C); the 99th percentile (30 °C); the maximum value of minimum temperatures (32 °C) and the minimum of daily minimum temperatures a (–6 °C). It is clear from this figure that the RR of preterm birth is significantly greater than 1 for the minimum temperature (–6 °C) at both lower and higher lags. 

**Figure 4:**
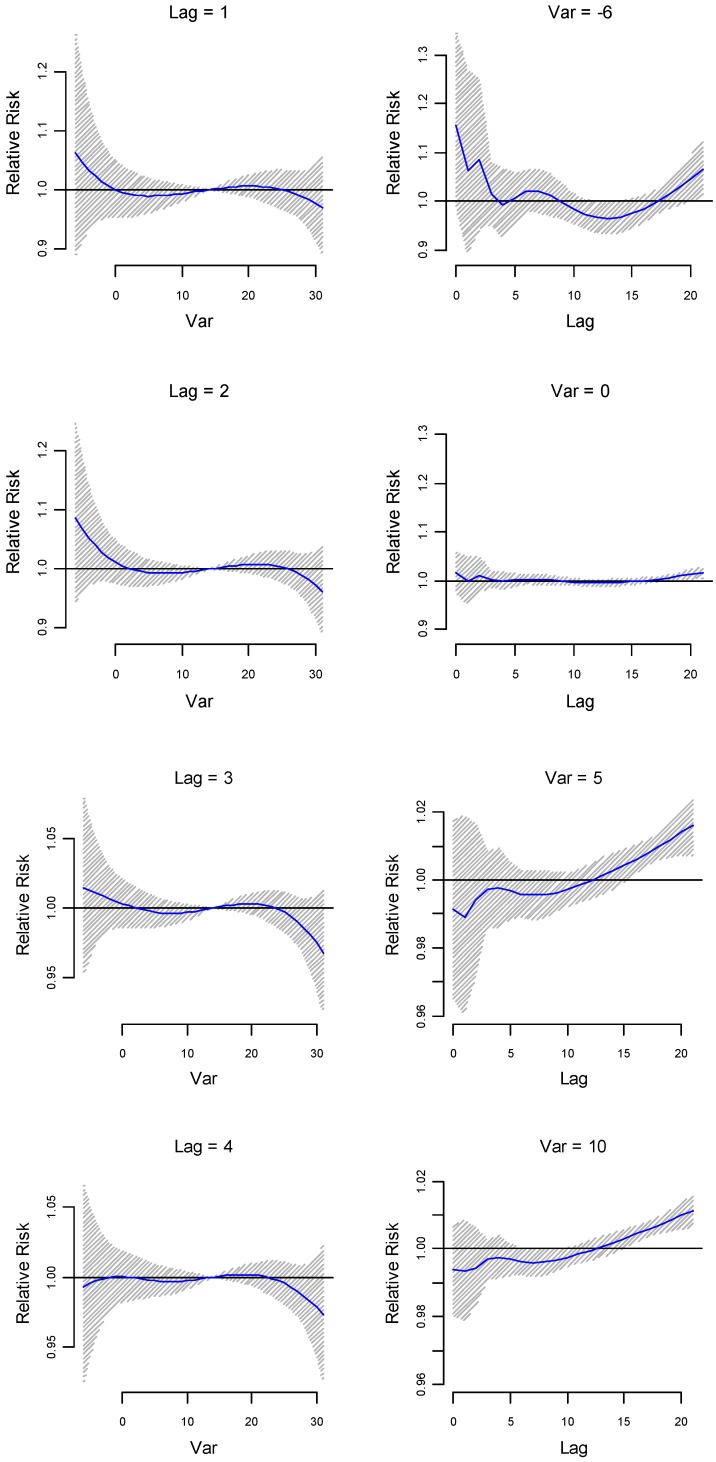
Lag-specific relative risk (RR) (at lower lags 1, 2, 3 and 4) estimates with their 95% confidence interval of preterm birth in different minimum temperatures (at –6 °C, 0 °C, 5 °C and 10 °C) using as a reference the median value of the whole annual series at 14 °C.

Lag-specific relative risk estimates (at lower lags 1, 2, 3 and 5) for different minimum temperatures (–6 °C, 0 °C, 5 °C and 10 °C respectively) are plotted in Figure 4. Immediate effects on relative risk estimates are observed for lower temperatures (21 °C to –6 °C). Delayed effects are also observed for lower temperatures (e.g., temperatures lower than 10 °C). While the effect of minimum temperature on risk of preterm birth is also significant (Figure 3a,b, and Figure 4), the effect appeared smaller than that of the maximum temperature (see Figure 1a,b and Figure 2).

The cumulative RRs for 21 days before delivery for minimum temperature was statistically significant for temperatures between –6 and 0 °C (see Figure S4b). An effect of the exposure to temperatures at 0 °C and –6 °C cumulatively up to 21 days before delivery was observed with an increased risk of preterm birth up to 7.2% (95% CI: 1.018 to 1.130) and 36.7% (95% CI: 1.023 to 1.824) respectively.

## 4. Discussion

In our study that focused on Alice Springs in Central Australia, a location which has extreme hot weather conditions and a high preterm delivery rate relative to the rest of Australia, we found that extreme hot and cold temperatures increase the relative risks of preterm birth. In the Poisson Generalised Additive Model run with the whole dataset (Indigenous and non-Indigenous), temperature dominates other explanatory variables such as Indigenous status and maternal age. Daily maximum temperatures and age of mothers were significant when the model was run using only the Indigenous population data. Maximum temperatures, anaemia, gestational diabetes and maternal age were significant when the model was run using the non-Indigenous pregnant mother dataset. The significant effect of maximum temperature is of particular relevance to health policy as Alice Springs is projected to become warmer as the climate changes. 

Our findings are consistent with that of several other studies that have observed an association between temperature extremes (extremely hot and cold days) and shorter gestational deliveries [9,12,13,14,15,16,17]. An immediate effect of the exposure to elevated temperature was observed with an increased risk of preterm birth of 1% to 2% when the maximum temperature was ≥41 °C. Immediate effects of exposure have also been observed in several studies [9,13,28]. Temperature exposures above 41 °C around 2–3 weeks before delivery show exponential increase in the risk of preterm birth. This also indicates delayed effects of temperature exposure.

While the analyses show that cumulative effects of exposure to temperature >40 °C for 3 weeks before delivery can increase the relative risks by around 8%, such extreme temperature events have not yet been recorded for Alice Springs. Recently, i.e., in February 2015, 21 days with a temperature maximum of >35 °C was recorded for Alice Springs. It has to be noted that while temperatures may not stay above 35 °C continuously across all the summer months, the majority of the days during summer have a temperature >35 °C. Consider that a heatwave is defined as days with temperature >40 °C consecutively for 3 days. If we assume linear increment of relative risk across days, then the increased relative risk for such events should be around 1.14%. While such events have not been recorded in historical data, such future events could occur in Alice Springs. The annual average number of days over 35 °C in Alice Springs is projected to increase from a current value of 89 to somewhere between 96 and 125 days by 2030 [27]. If such hot periods become more extreme with temperatures becoming >40 °C, relative risks of the exposure will be more substantial compared to the current risks. While we focus on the cumulative effects up to 3 weeks before delivery, the effects of shorter duration heatwave events in Brisbane have also been observed to have an effect on preterm delivery [23]. The cumulative effects of minimum temperatures (–6 to 0 °C) are much higher (36.2 to 7.2% increased relative risk), but such events have not been recorded for Alice Springs and are likely not to happen in the near future due to climatic changes. In 2015, there were two events where there were 3 days with temperatures consecutively under –2 °C. The cumulative effect of days with temperatures under –2 °C for 21 days is 13% increased relative risk. Again assuming a linear increment, the increased relative risk for a 3 days event will be around 1.9%. The cumulative effects of temperature exposure for the non-Indigenous population follow a similar pattern, but the relative risks are much higher. On the other hand, the cumulative risks for the Indigenous population increase by up to 12% between 30 and 38 °C, but interestingly decreases beyond 38 °C. A number of reasons could be attributed to this. This could be because (i) Indigenous communities remain in the outdoor environment at temperatures between 30 and 38 °C and might gradually acclimatise to the increasing temperatures during the summer periods or (ii) beyond particular threshold temperatures they may adopt certain measures (e.g., reducing outdoor time) to adapt to the extreme hot weather. Further investigation is required to explore the effect of temperature on Indigenous pregnant mothers. 

Immediate risks to preterm birth is also observed for cold temperatures, with an increased risk of more than 10% observed for extremely cold temperatures such as –6 °C. Lower immediate risks are observed for temperatures between –6 °C and 26 °C. The immediate increase in relative risk due to the extreme cold temperature recorded (–6 °C) is higher than that of the extreme hot temperature recorded (45 °C). The effects of minimum temperatures are likely to be milder for Alice Springs in the future, given that minimum temperatures are projected to increase under climate change. Delayed effects are observed for both minimum and maximum temperature exposures as observed in [28]. 

The warmer temperature projections for locations in Central Australia would mean that the relative risk of preterm birth is likely to be higher in the future. This is of particular relevance to health practitioners as they need to create awareness among pregnant women to reduce their exposures to extreme heat during the summer months and take necessary precautionary actions. While this study focuses only on Alice Springs, it has relevance internationally as we anticipate similar results in other hot desert towns. 

The analysis conducted in this paper is limited by (i) the small number of births in Alice Springs; (ii) the availability of data for other confounding variables; (iii) inadequate information in the NT Midwives dataset we had obtained; and (iv) model design. Firstly, the dataset analysed is quite small compared to many recent studies that were conducted in New York [18], China [31], Spain [28], Brisbane [23] and Rome [16]. Alice Springs is a small town, with average births around 1.65 per day at the Alice Springs Hospital. The study still has its importance, given that most of the other studies were conducted in temperate and tropical climates and mainly in cities. 

In the NT Midwives Data Collection, there were no details on medications provided to delay labour. Early labour could be prolonged through medication, but the available data set does not account for that, so the potential number of natural pre-term births could be underestimated. The methods used to calculate gestation age (e.g., ultrasound, last menstrual period date) may have different accuracies. The exact methods used for determining the gestational age were unknown. Also, gestational ages were approximated as whole weeks and not available as days and weeks in the dataset. Alcohol consumption and smoking during pregnancy have been found to have an effect on preterm birth in other studies [32,33]. It is difficult to conclude the same in this study due to the large number of “not stated” options that were found under the variables “alcohol use” (~43%) and “smoking” (45%). Also, only 7% and 16% of the sample recorded alcohol consumption and smoking, respectively, during pregnancy. Air pollution data was also not available for Alice Springs and hence its effects remain unknown. While associations between stillbirths and temperature has been explored in some studies [34,35], it was not analysed in this study due to limited data (only 1% of the data in the Alice Springs Hospital was stillborn during the period considered). 

Time of delivery was not available in the dataset. A more accurate analysis would have required time of delivery and hourly temperature data, especially because immediate increase to the RR of preterm birth was observed in the study. Temperature data used in the analysis originates from the Alice Springs airport station, which is around 10 km away from the town and hence may not precisely represent the temperature conditions of the Alice Springs town area.

In the absence of any complications, pregnant women from far remote communities are requested to arrive and stay in a resident hostel in Alice Springs around 6 weeks before their due date. This would mean some of the preterm deliveries in Alice Springs Hospital may have been exposed to different temperature and humidity conditions. The model was designed to understand the effect of the temperature variables up to 3 weeks before delivery. The analysis clearly shows that delayed effects are observed within the three week period, and hence examining the effects of temperature beyond three weeks may also provide more insights. However, then spatial variation of temperature will have to be accounted for in the case of pregnant women who travel from remote locations. 

## 5. Conclusions

In conclusion, the study summarises that extreme maximum and minimum temperatures increase the risk of preterm birth and the effects are influenced by the values of the temperature. The effects of pollution, which may be significant, could not be considered due to data unavailability. Air quality monitoring, more precise temperature measurements and analysis on longer term temperature exposures are required. The main finding from this study is that pregnant women may have to reduce exposure to extreme temperatures in Central Australia. Awareness on the potential risks of exposure to extreme hot and cold conditions and recommendations on how to reduce their effect should be disseminated through health care organisations. A range of behavioural responses, environmental modifications and technological adaptation may also be required to reduce the effects of temperature exposure to pregnant women. 

## Supplementary Materials

The following are available online at www.mdpi.com/1660-4601/14/2/147/s1, Figure S1: Block diagram showing how the final dataset was obtained from the original source (Department of Health Gains Planning Unit, NT Department of Health), Figure S2: Monthly average of the total births (a) and the autocorrelation plot of seasonally differencing data (b), Figure S3: RR of preterm birth for the exposure to different maximum temperatures for Indigenous data (a) and non-Indigenous data (b). The median value of the whole temperature annual series (30 °C) is used as reference, Figure S4: Cumulative relative risk estimates for 21 days with their 95% confidence intervals for different maximum (a) and minimum temperatures (b). The reference temperature is the median maximum temperature value of the summer season, 30 °C, Figure S5: Cumulative relative risk estimates for 21 days with their 95% confidence intervals for different maximum temperatures using Indigenous data (a) and non-Indigenous data (b). The reference temperature is the median maximum temperature value of the summer season, 30 °C, Table S1: Descriptive summary of temperature data, Table S2: Descriptive summary of the Alice Springs Hospital data used for analyses.
