# Supplementary material for: Examining the Effects of Ambient Temperature on Pre-Term Birth in Central Australia
Source: Int J Environ Res Public Health. 2017 Feb 4;14(2):147. doi: 10.3390/ijerph14020147 (PMC5334701; doi:10.3390/ijerph14020147)
Supplement: Supplementary file 1 [file ijerph-14-00147-s001.pdf]

Supplementary Materials: Examining the Effects of Ambient Temperature on Pre-Term Birth in Central Australia

Supriya Mathew, Deepika Mathur, Anne B. Chang, Elizabeth McDonald, Gurmeet R. Singh, Darfiana Nur and Rolf Gerritsen

Table S1. Descriptive summary of temperature data.

Meteorological variables	Complete Annual Series °C		Summer Season (December–February) °C				
	Median	Minimum	Median	Percentiles			Maximum
				90th	95th	99th	
Maximum temperature	30	7	36.70	40.90	41.80	43.20	45
Minimum temperature	14	-6	21.40	25.90	27.30	29.50	31.70
Average temperature	22.30	5	29.10	33	33.90	35.6	37.10

Source Bureau of Meteorology Alice springs airport station 15590 downloaded via http://www.bom.gov.au/climate/data/ for the period 1986–2013.

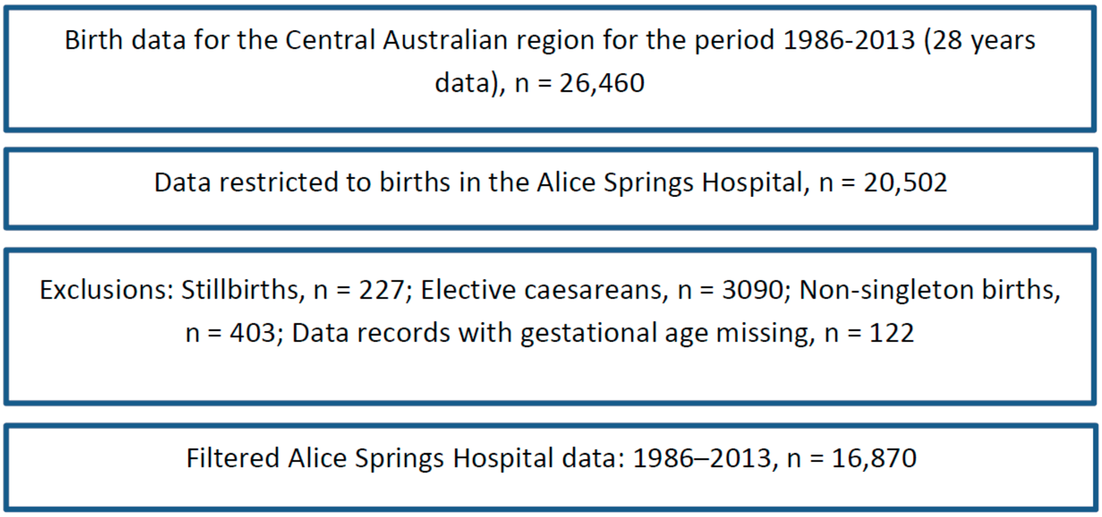

Figure S1. Block diagram showing how the final dataset was obtained from the original source (Department of Health Gains Planning Unit, NT Department of Health).

Table S2. Descriptive summary of the Alice Springs Hospital data used for analyses.

Data Characteristics		ASH Data Used n = 16,870	ASH Pre Term Births n = 1401
Sex	Male	8726	729
	Female	8136	671
	Unknown	8	1
Birth weight (Average in kg)		3.30	2.48
Maternal age (Average in years)		26	25
Indigenous status	Indigenous	7996	951
	Non-indigenous	8873	450
	Not stated	1	0
Gestation age (Average in weeks)		38.9	34.3

S.1. Analysis of Birth Seasonality

Seasonal variation of monthly total births and monthly average of total births was checked using the plots of autocorrelations (see Figure S2). The Figure shows that data may contain polynomial trends, non-constant variation and seasonality. Using the autocorrelation function plots of seasonal differencing for each time series, we confirmed the non-stationarity and seasonality in the data. The seasonality is apparent for each series as the autocorrelation function is significant at lags = 12 with peaks in birth observed around March and April every year.

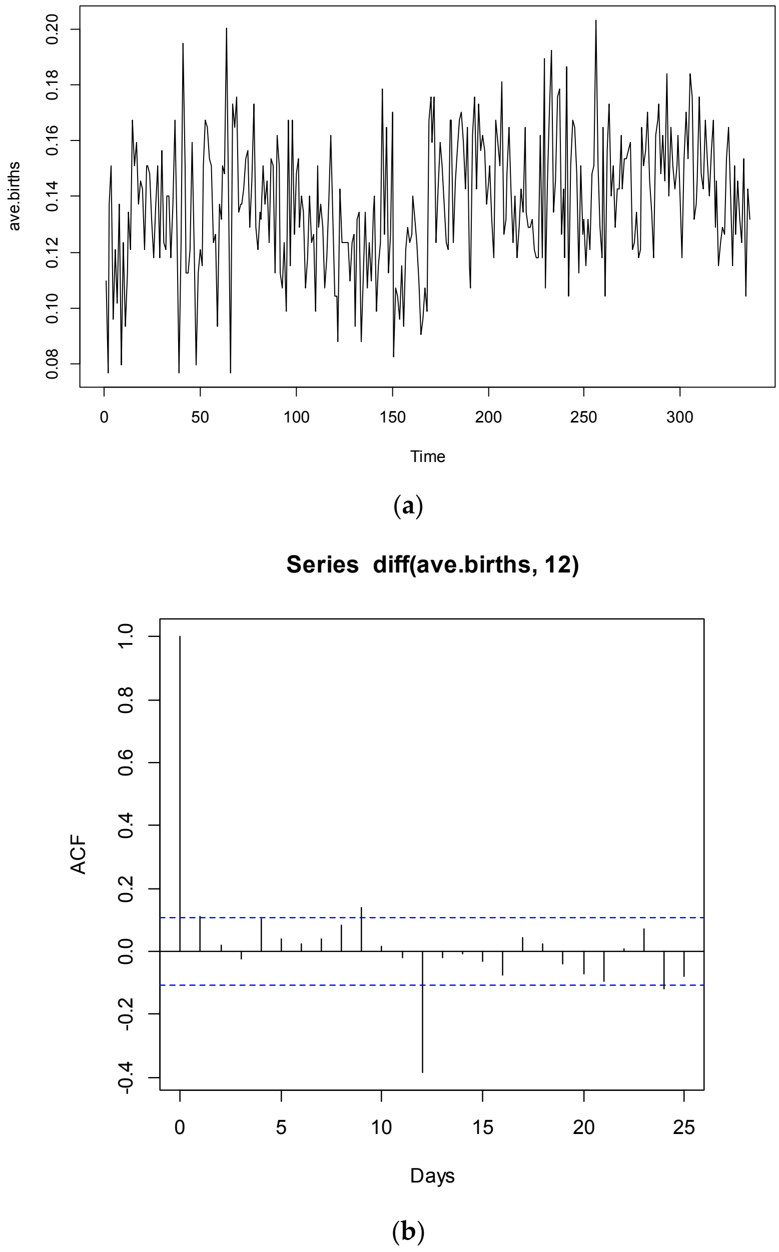

Figure S2. Monthly average of the total births (**a**) and the autocorrelation plot of seasonally differencing data (**b**).

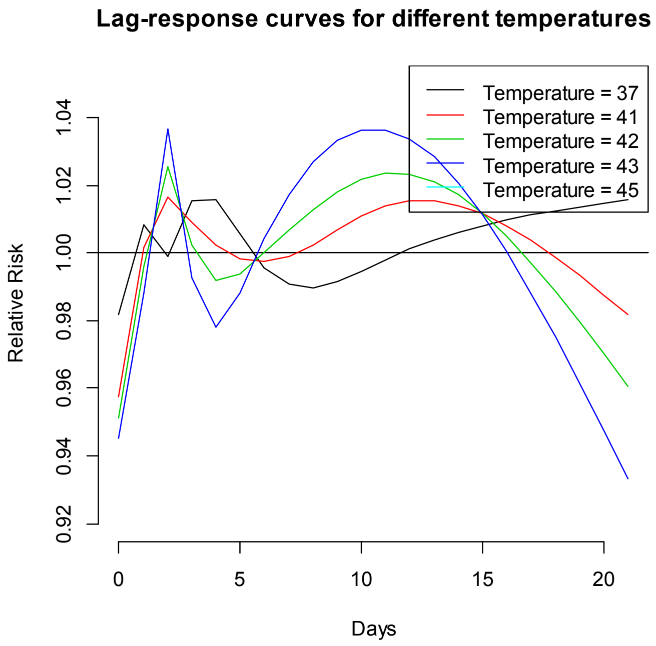

(**a**)

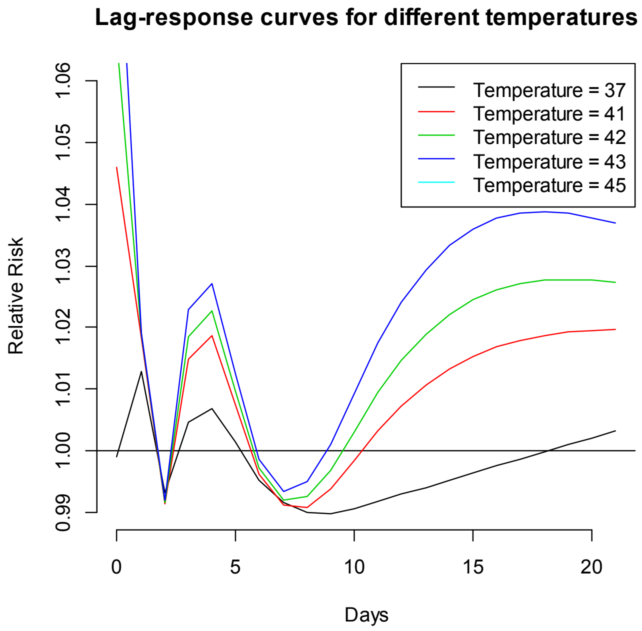

(**b**)

Figure S3. RR of preterm birth for the exposure to different maximum temperatures for Indigenous data (**a**) and non-Indigenous data (**b**). The median value of the whole temperature annual series (30 °C) is used as reference.

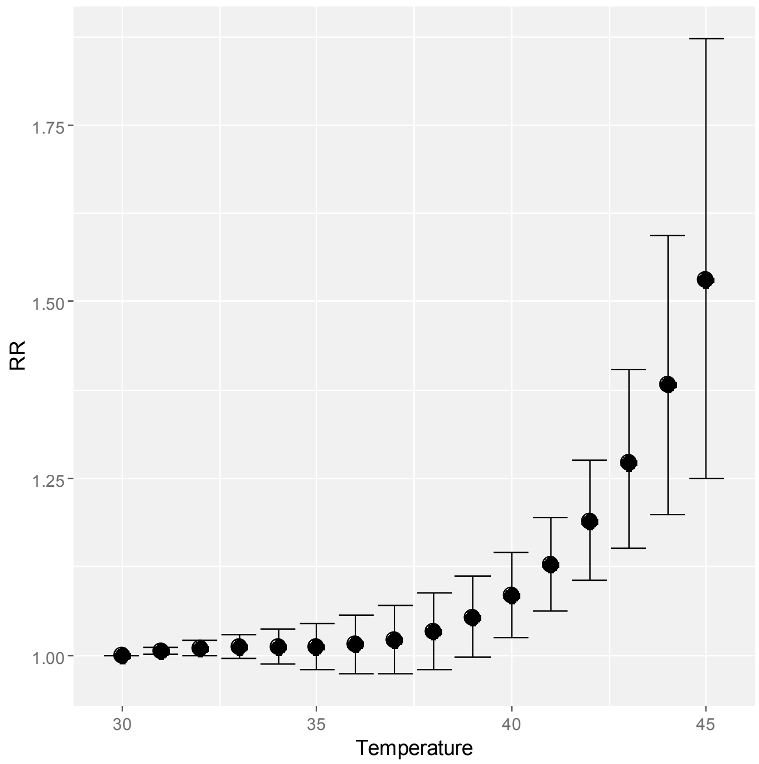

(**a**)

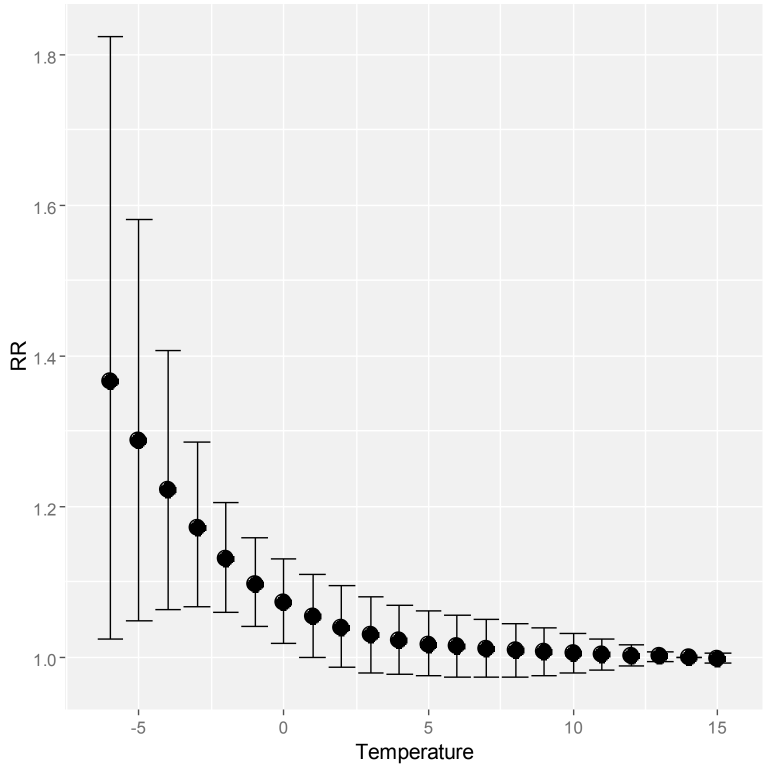

(**b**)

Figure S4. Cumulative relative risk estimates for 21 days with their 95% confidence intervals for different maximum (**a**) and minimum temperatures (**b**). The reference temperature is the median maximum temperature value of the summer season, 30 °C.

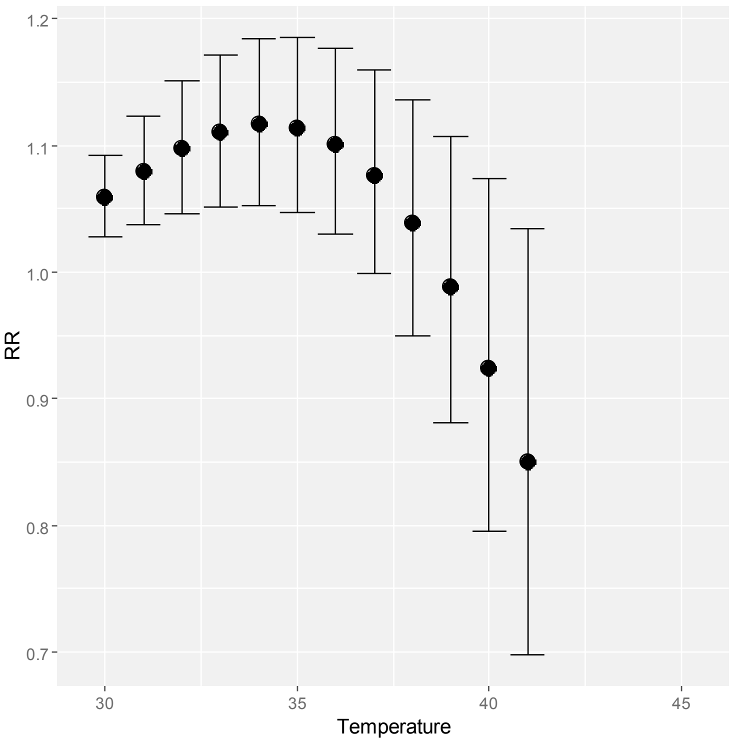

(**a**)

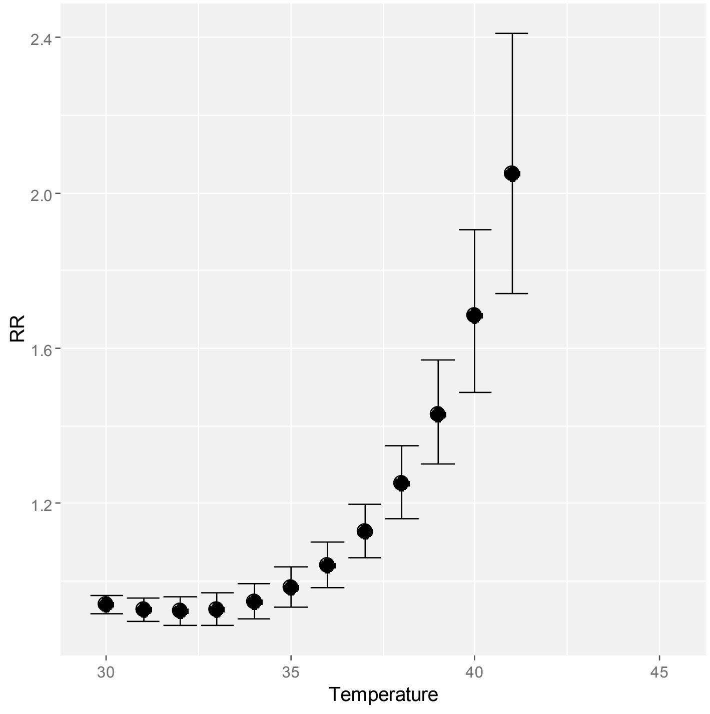

(**b**)

Figure S5. Cumulative relative risk estimates for 21 days with their 95% confidence intervals for different maximum temperatures using Indigenous data (**a**) and non-Indigenous data (**b**). The reference temperature is the median maximum temperature value of the summer season, 30 °C.

© 2017 by the authors; licensee MDPI, Basel, Switzerland. This article is an open access article distributed under the terms and conditions of the Creative Commons by Attribution (CC-BY) license (http://creativecommons.org/licenses/by/4.0/).
